# Sporadic Hemangioblastoma of the Kidney: a rare renal tumor

**DOI:** 10.1186/1746-1596-7-49

**Published:** 2012-05-01

**Authors:** Yang Liu, Xue-shan Qiu, En-Hua Wang

**Affiliations:** 1 Department of Pathology, the First Affiliated Hospital and College of Basic Medical Sciences, China Medical University, Shenyang, 110001, China; 2 Institute of Pathology and Pathophysiology, China Medical University, Shenyang, 110001, China

**Keywords:** Hemangioblastoma, Von Hippel-Lindau disease, Renal cell carcinoma, Epithelioid hemangiopericytoma, Epithelioid angiomyolipoma

## Abstract

**Abstract:**

Hemangioblastoma is a benign and morphologically distinctive tumor that can occur sporadically or in association with von Hippel-Lindau disease in approximately 25% of the cases, and which involves the central nervous system in the majority of the cases. Rare occurrences of hemangioblastoma in peripheral nerves and extraneural tissues have been reported. This report describes one case of sporadic renal hemangioblastoma happened in a 16-year-old Chinese female patient, presenting with hematuria, and low back pain. Histologically, the tumors were circumscribed, and composed of sheets of large polygonal cells traversed by arborizing thin-walled blood vessels. The diagnosis of hemangioblastoma was confirmed by negative immunostaining for cytokeratin, and positive staining for α-inhibin, S100 and neuron-specific enolase (NSE). This benign neoplasm which can be mistaken for various malignancies such as renal cell carcinoma, epithelioid hemangiopericytoma and epithelioid angiomyolipoma, deserves wider recognition for its occurrence as a primary renal tumor.

**Virtual slides:**

The virtual slide(s) for this article can be found here: http://www.diagnosticpathology.diagnomx.eu/vs/5445834246942699

## Background

Hemangioblastoma, also known as capillary hemangioblastoma, is a benign tumor of uncertain histogenesis, consisting of networks of small blood vessels interspersed with lipid-laden stromal cells [1]. Although the stromal cells are often bland-looking, they can exhibit significant nuclear pleomorphism, mimicking carcinoma, or other malignancies. Hemangioblastoma occurs sporadically, or in a setting of von Hippel-Lindau disease in approximately one-quarter of the cases [2]. This tumor typically occurs within the central nervous system, predominantly in the cerebellum, but also occasionally in the meninges, retina, spinal cord, corpus callosum, lateral ventricle, pituitary gland, and the optic nerve. Exceptionally, hemangioblastoma occurs in other sites, such as peripheral nerve, soft tissue, retroperitoneum, skin, liver, pancreas, lung, adrenal, kidney, and urinary bladder, usually in the setting of known von Hippel-Lindau disease. There are only a few reports on sporadic hemangioblastoma occurring outside the central nervous system, including kidney [3]. We report one such case involving the kidney, which might be mistaken for other renal tumors, in particular clear cell renal cell carcinoma.

## Case presentation

### Clinical History

A 16-year-old Chinese female, presented with gross hematuria and low back pain for 18 months. A homogeneous solid mass in the upper pole of the left kidney was detected on a computed tomographic scan. Nephrectomy was performed, showing a 1.2 cm well-encapsulated brownish-white tumor with some hemorrhagic areas in the upper pole of left kidney. There was no polycythemia or clinical evidence of von Hippel-Lindau disease. At imaging, no other tumor was detected, particularly in the central nervous system (CNS). The patient was alive with no tumor recurrence or metastasis at half a year of follow-up.

### Gross features

The left kidney was normal in size (12.0 × 5.0 × 4.0 cm). A 1.2 cm well-encapsulated mass was found in the upper pole of left kidney. The cut surface of the tumor was brownish-white, solid and homogeneous.

### Microscopic features

**Figure 1:**
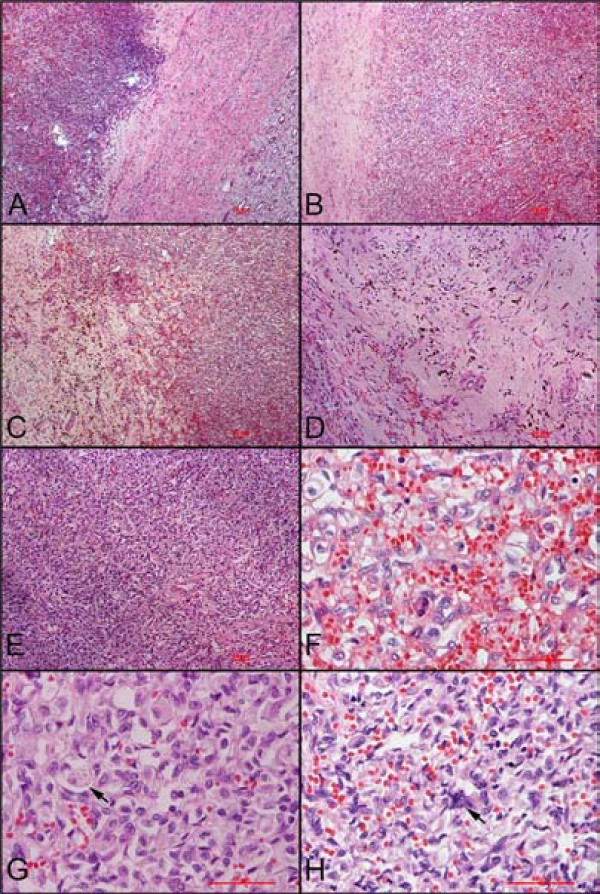
**Histological features of this case**. A and B: The tumor was surrounded by a thick fibrous capsule and well-demarcated from the surrounding renal parenchyma. C: It was characterized by an alternation of cellular and paucicellular areas. D: The paucicellular areas were mainly composed of fibrous stroma containing reticular vascular channels, hemosiderin pigment, and rare stromal cells. E: The cellular areas were composed of stromal cells surrounded by a rich capillary network. F and G: The cytoplasm of stromal cells is weakly eosinophilic, and locally contains lipid droplets (arrow) but no hyaline globules. And the stromal cells also showed ovalnuclei, delicate chromatin, and inconspicuous nucleoli. H: Occasionally, pleomorphic, or bizarre tumor cell nuclei was seen.

The tumor was characterized by an alternation of cellular and paucicellular areas surrounded by a thick fibrous capsule and well-demarcated from the surrounding renal parenchyma (Figure 1A-E). The paucicellular areas were mainly composed of fibrous stroma containing reticular vascular channels, hemosiderin pigment, and rare stromal cells (Figure 1D). The cellular areas were composed of a rich capillary network of single-layered flat endothelial cells enclosing stromal cells. These last ones showed a pale or eosinophilic cytoplasm exhibiting occasional lipid droplets but no hyaline globules, ovalnuclei, delicate chromatin, and inconspicuous nucleoli (Figure 1F-G). Pleomorphic, or bizarre tumor cell nuclei was barely seen (Figure 1H), and no necrosis or mitoses was found.

### Immunohistochemistry

**Figure 2:**
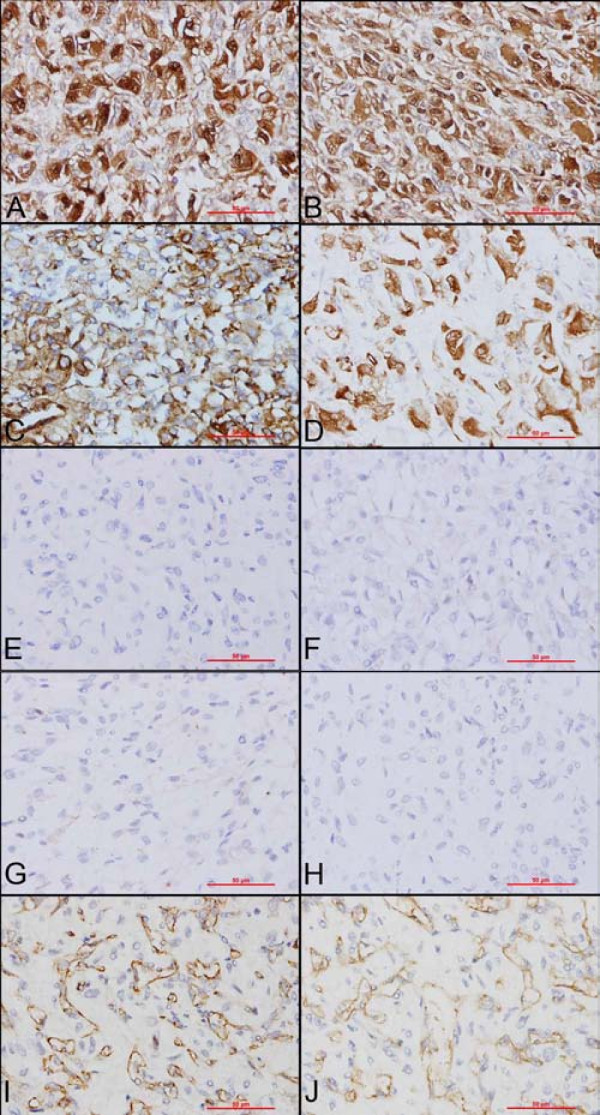
**Immunohistochemical staining**. A: Positive staining for NSE. B: Positive staining for S100 protein. C: Positive staining for vimentin. D: Positive staining for α-inhibin. E: Negative staining for epithelial membrane antigen (EMA). F: Negative staining for AE1/AE3. G: Negative staining for CD10. H: Negative staining for HMB-45. I and J: CD34 and CD31 underlined the rich and delicate vascular channels, whereas the tumor cells were negative.

The immunohistochemical study showed that stromal cells were diffusely positive for NSE (Figure 2A), S-100 protein (strong cytoplasmic and nuclear staining, Figure 2B), and vimentin (Figure 2C). Stromal cells expressed more focal α-inhibin (Figure 2D). They were strictly negative for epithelial membrane antigen (EMA), AE1/AE3, CD10, and HMB-45 (Figure 2E-H). The other markers studied were strictly negative (melan-A, CD56, chromogranin, synaptophysin, calretinin, smooth muscle actin, and desmin). Finally, CD34 and CD31 underlined the rich and delicate vascular channels (Figure 2I, J). All of the immunohistochemical results were summarized in Table 1.

**Table 1:** Panel of immunohistochemical stains

**Immunohistochemical Stain**	**Result**
Pan-cytokeratin (AE1/AE3)	-
epithelial membrane antigen (EMA)	-
vimentin	+
α-inhibin	+
Neuron-specific enolase (NSE)	+
S100 protein	+
Synaptophysin	-
Chromogranin	-
HMB-45 (melanoma-associated marker)	-
melan-A	-
Calretinin	-
smooth muscle actin	-
Desmin	-
CD10	-
CD34	-
CD31	-

## Discussion

Because of the few reports of sporadic primary hemangioblastoma, the progression and prognosis of this tumor was still unclear. To our knowledge, there are five cases of hemangioblastoma involving the kidney which have been reported [4,5]. Hemangioblastoma is likely to be an underrecognized tumor of the kidney, because it mimics many tumor types morphologically and it is usually not considered in the differential diagnosis. A correct diagnosis is important because hemangioblastoma is benign even if there are highly atypical tumor cells, and the patient should be evaluated for possible von Hippel-Lindau disease. Because of the prominent vasculature and large neoplastic cells with atypical nuclei, renal hemangioblastoma can be mistaken for many renal neoplasms including renal cell carcinoma (RCC) and epithelioid angiomyolipoma, adrenal cortical carcinoma and paraganglioma (pheochromocytoma). Sometimes, it can also be mistaken for other rare tumors in this location as epthelioid hemangiopericytoma or lobular capillary hemangioma.

Although renal hemangioblastoma mimics various renal neoplasms, it can be recognized or suspected on morphologic grounds. The clues to the diagnosis are: circumscribed borders, paucity of mitotic figures despite prominence of atypical cells, fine vacuoles in some tumor cells indicating presence of intracytoplasmic lipids, and rich capillary network with focal pericytomatous pattern.

In this case, all the features described above were observed except for fine cytoplasmic vacuoles, so the first diagnosis came to our mind was epithelioid hemangiopericytoma instead of hemangioblastoma. Because of numerous variants of RCC have been described in recent years which markedly expanding the morphologic spectrum and rendering it difficult to totally rule out RCC on morphologic grounds. Among the variants of RCC, clear cell RCC is the main differential diagnosis as it shares several morphologic features with hemangioblastoma. This tumor can have occasionally a hemangioblastoma-like pattern which makes it nearly impossible to distinguish with hemangioblastoma on morphologic grounds. In such particular case, immunohistochemical staining might be the only solution. In contrast to hemangioblastoma, clear cell RCC is usually negative for α-inhibin, S100, and NSE and positive for AE1/AE3, EMA and CD10 [5,6]. And in this case, the tumor cells show striking morphologic mimicry of epithelioid angiomyolipoma. The epithelioid tumor cells of the latter often shows homogeneous or reticulated (spidery) cytoplasm, sometimes with grumous basophilic material, instead of lipid-containing vacuolated cytoplasm. Fat cells and thick-walled blood vessels with spindly cells radiating from the wall, if present, provide further support to the diagnosis. In addition, melanin marker (HMB45 or melan-A) and muscle marker (smooth muscle actin or desmin) will be helpful to diagnosis.

Adrenal cortical carcinoma may directly invade or metastasize to the kidney. But the tumor cells commonly show lipid cytoplasmic vacuoles; mitotic activity, infiltrative growth and vascular invasion are often identifiable. In addition, immunohistochemical staining will be helpful to further demonstrate. Paraganglioma (pheochromocytoma) often shows a definite nested pattern in at least some foci which was hardly seen in this case.

To our surprise, the immunophenotype (CD34^-^, melan-A^-^, HMB-45^-^, smooth muscle actin^-^, muscle specific actin^-^ and desmin^-^) overthrows the diagnosis of epithelioid hemangiopericytoma or angiomyolipoma. Negative staining for AE1/AE3, EMA and CD10 exclude the probability of RCC; Negative staining for synaptophysin, chromogranin and calretinin exclude the probability of adrenal cortical carcinoma and paraganglioma. In addition, S100 was diffuse positive in this case, while in paraganglioma, S100+ sustentacular cells are often found surrounding the nests of tumor cells. All of the diagnosises were overthrowed by immunohistochemical staining, so we searched the similar case on PubMed ( http://www.ncbi.nlm.nih.gov). After we learned that sporadic hemangioblastoma might happen in this location, we reviewed this case carefully and found only a few lipid droplets and bizarre tumor cell nuclei in tumor cytoplasm which was indicated by arrow in Figure 1G-H. NSE and α-inhibin were also added to stain. The immunophenotype (NSE^+^, S100^+^ and α-inhibin^+^) also demonstrated the diagnosis of hemangioblastoma. Furthermore, more cytoplasmic multiple sharply delineated vacuoles were observed in the slides performed for immunohistochemistry (Figure 2A, B, and D). The morphologic characteristics of this case were strikingly superimposable to those previously described in this location, including the clues to the diagnosis defined by Ip et al. [4] The immunophenotypic profile (AE1/AE3-, S100+, NSE+, and α-inhibin+) reported by Ip et al [4] was also noted.

## Conclusions

In this case, the tumor was nearly mistaken for epithelioid hemangiopericytoma which indicates it’s hard to distinguish between them in the practical work. Cytoplasmic lipid droplet maybe indicates a diagnosis of hemangioblastoma, but don’t exclude the probability when it was hardly seen, especially in the unfamiliar renal tumors with abundant capillary network. The stromal cells may be or may not be atypical with bizarre nuclei. Therefore, hemangioblastoma must be included in the dfferential diagnosis of renal tumors to not underestimate this tumor in this location and so to better evaluate its real frequency and not establish wrongly a diagnosis of malignancy to this benign tumor. Using combination of immunohistochemistry may be helpful to some rare renal tumors.

## Competing interests

The authors declare that they have no competing interests.

## Authors' contributions

YL analyzed the data and wrote the manuscript as a major contributor. X-SQ and E-HW helped to revise the discussion section of this manuscript. All authors have read and approved the final manuscript.

## Consent

Written informed consent was obtained from the parents of the patient for publication of this case report and accompanying images. A copy of the written consent is available for review by the Editor-in Chief of this Journal.
